# Oral health in the Japan self-defense forces - a representative survey

**DOI:** 10.1186/1472-6831-11-14

**Published:** 2011-04-19

**Authors:** Yuka Kudo, Mike T John, Yoko Saito, Shachi Sur, Chisako Furuyama, Hiroaki Tsukasaki, Kazuyoshi Baba

**Affiliations:** 1 Department of Prosthodontics, Showa University, Japan; 2 Department of Diagnostic and Biological Sciences, University of Minnesota, USA

## Abstract

**Background:**

The oral health of military populations is usually not very well characterized compared to civilian populations. The aim of this study was to investigate two physical oral health characteristics and one perceived oral health measure and their correlation in the Japan self-defense forces (JSDF).

**Methods:**

Number of missing teeth, denture status, and OHRQoL as evaluated by the Japanese 14-item version of the Oral Health Impact Profile (OHIP-J14) as well as the correlation between these oral health measures was investigated in 911 personnel in the JSDF.

**Results:**

Subjects did not have a substantial number of missing teeth and only 4% used removable dentures. The mean OHIP-J14 score was 4.6 ± 6.7 units. The magnitude of the correlation between the number of missing teeth with OHIP-J14 scores was small (r = 0.22, p < 0.001). Mean OHIP-J14 scores differed between subjects with and without dentures (8.6 and 4.4, p < 0.001).

**Conclusions:**

Compared to Japanese civilian populations, personnel of the JSDF demonstrated good oral health. Two physical oral health characteristics were associated with perceived oral health.

## Background

In many countries oral health of the general population and of various patient populations is well characterized. However, in military populations it is often not characterized even if oral health is considered an important part of general health and therefore influences the health status of military personnel and their ability to perform their duties. Furthermore, their good oral health would decrease the number of urgent dental interventions and absences from training and the battlefield that would in turn, improve the security of the whole formation [1].

Oral health has two dimensions. First, there is the physical oral health status in terms of number of teeth, periodontal status, mouth opening etc. Second, how the individual perceives his or her oral health is equally important. Both dimensions are needed to characterize oral health comprehensively.

Key characteristics of physical oral health are the number of teeth and denture status and such findings are standard components of oral health surveys for non-military populations [2-5]. For example, one study that investigated a general population, age ranging 20 to 59 years old, reported that 49.0% of the subjects had intact dental arch with no missing teeth, while 39.0% had 1 to 4 and 12.1% had 5 or more missing teeth and that the majority of these subjects with missing teeth had fixed partial dentures and only a small number of subjects had removable partial dentures (7%) [6].

The most comprehensive concept describing perceived oral health is oral health related quality of life (OHRQoL), which has been recognized more and more frequently as an important component of health [7]. Therefore, collecting OHRQoL information in oral health surveys is increasingly performed to provide complementary information in addition to physical oral health indicators [8-10]. One of the instruments frequently used to measure OHRQoL is the Oral Health Impact Profile (OHIP) questionnaire [7], which asks about the impact of oral conditions on everyday well-being. The OHIP questionnaires have been translated into various languages in both full and abbreviated versions, ranging from 49 to 5 items [7,11-14]. A Japanese long version (OHIP-J54) [15] and a short version (OHIP-J14) [16] have recently been developed.

For military personnel's oral health, some reports are available in the literature [1,17,18]; however no data has been published so far for the Japan self-defense forces.

The aim of this study was to investigate two physical oral health characteristics (number of missing teeth and denture status) and one perceived oral health measure (oral health related quality of life, OHRQoL) and their correlation in the Japan self-defense forces (JSDF).

## Methods

Subjects were consecutively sampled from a Camp of the JSDF during the annual medical examination in 2008 (mean aged 35.7 ± 10.1, range 15-59 years). Nine hundred and eleven individuals agreed to participate in this study and gave informed consent.

To assess physical oral health, tooth status of the participants was examined by a single dentist and recorded as present or absent. For analysis, missing teeth were categorized (0, 1, or 2+), while the fixed prostheses were regarded as the teeth were present. Denture status was categorized as fixed or removable prostheses.

To assess perceived oral health, oral health-related quality of life was evaluated using OHIP-J14 [16]. For each of the 14 OHIP questions, subjects were asked how frequently they had experienced impact in the preceding 12 months and coded as 4 = very often, 3 = fairly often, 2 = occasionally, 1 = hardly ever and 0 = never. The responses were summed up into a score ranging from 0 to 56. A score of 0 indicated no perceived oral health problem and 56 indicated maximum impairment.

To measure how physical and perceived oral health are related, a Pearson correlation coefficient assessed the correlation between number of missing teeth and OHIP-J14 scores and a point-biserial correlation coefficient assessed the correlation between denture status and OHIP-J14 scores. Subgroup correlation analyses were performed in 4 age groups and gender. A t-test analyzed the difference in the mean OHIP-J14 scores between subjects with and without removable partial dentures.

The study protocol was conducted in compliance with the Helsinki Declaration and was approved by the Ethics Committees of Showa University (#2007-29).

## Results

### Subject characteristics

Subjects had a mean age of 35.7 years (SD: 10.1, min: 15, max: 59 years) and were predominantly male (Table 1).

**Table 1:** Demographics

Characteristics	N	%
Age		
15-24 yrs	161	17.7
25-34 yrs	284	31.2
35-44 yrs	258	28.4
45-59 yrs	207	22.8
Females	60	6.6

### Number of teeth and denture status

The subjects did not have many missing teeth [mean: 0.85, prevalence of subjects with at least one (and not with fixed partial dentures replaced) missing tooth: 28%]. The majority of subjects (N = 678, 74%) had neither fixed nor removable dentures. Among the remaining 233 subjects, only 38 subjects had removable partial dentures and one subject had complete dentures, totaling 4% of the whole sample (Table 2). The data form the subject with complete dentures was integrated in the group of removable dentures for the analyses. Among age and gender characteristics, age was the strongest factor differentiating tooth loss and denture status.

**Table 2:** Number of missing teeth and frequency of denture types for all subjects and stratified by age and gender

*characteristic*		*Missing teeth*				*Denture Status*	
		0	1	2+		No Denture	Removable Denture
				
	N				%		
all subjects	911	72.5	12.1	15.5		95.7	4.3
							
age groups							
15-24 yrs	161	88.8	4.4	6.8		100.0	0.0
25-34 yrs	284	83.1	9.9	7.0		99.6	0.4
35-44 yrs	258	62.8	16.7	20.5		95.7	4.3
45-59 yrs	207	57.5	15.0	27.5		87.0	13.0
							
male	850	72.0	12.6	15.4		95.0	5.0
female	60	80.0	3.3	16.7		95.8	4.2

### Oral health-related quality of life

The subjects did not suffer from major OHRQoL impairment. Only 0.2 to 1.9% reported frequent negative impacts (response categories fairly often or very often) with mean scores for those items ranging from 0.24 to 0.41 (Table 3). The most commonly reported impact was within the dimension of 'physical discomfort', 1.9% reported being self-conscious fairly often or very often. The mean OHIP score for all subjects was 4.6 +/- 6.7 (95% confidence interval = 4.1 - 5.0, Table 4).

**Table 3:** Distribution of responses (%)

Dimension and description of item 'Because of problems with your teeth, mouth or dentures, during the last 1 months,...'	Never(0)/hardly ever(1)	Occasionally (2)	Fairly often(3)/very often(4)	Mean
**Functional limitation**				
Have you had trouble pronouncing any words?	94.4	4.6	1.0	0.3
				
Have you felt that your sense of taste has worsened?	97.3	2.3	0.4	0.2
				
**Physical pain**				
				
Have you had painful aching in your mouth?	92.6	6.2	1.2	0.4
				
Have you found it uncomfortable to eat any foods?	92.7	5.9	1.4	0.4
				
**Psychological discomfort**				
				
Have you been self-conscious?	90.5	7.6	1.9	0.4
				
Have you felt tense?	93.5	5.7	0.8	0.4
				
**Physical disability**				
				
Has your diet been unsatisfactory?	95.5	4	0.5	0.3
				
Have you had to interrupt meals?	97.5	2.3	0.2	0.2
				
**Psychological disability**				
				
Have you found it difficult to relax?	93.4	5.1	1.5	0.3
				
Have you been a bit embarrassed?	95.0	4.1	0.9	0.4
				
**Social disability**				
				
Have you been a bit irritable with other people?	93.7	5.1	1.2	0.4
				
Have you had difficulty doing your usual jobs?	96.7	2.8	0.5	0.3
				
**Handicap**				
				
Have you felt that life in general was less satisfying?	94.7	4.0	1.3	0.4
				
Have you been totally unable to function?	97.0	2.3	0.7	0.3

**Table 4:** OHIP-J14 summary score for all subjects and stratified by age and gender

*Characteristic*		*OHIP mean (SD)*	*95% confidence interval for the mean*
all subjects		4.6 (6.7)	4.1 - 5.0
			
age groups			
15-24 yrs	161	4.1 (6.9)	3.0 - 5.1
25-34 yrs	285	3.0 (5.3)	2.4 - 3.6
35-44 yrs	258	5.3 (6.7)	4.4 - 6.1
45-59 yrs	207	6.1 (7.6)	5.1 - 7.1
			
Males	851	4.5 (6.7)	4.1 - 5.0
Females	60	4.9 (6.8)	3.1 - 6.6

### Correlation between physical characteristics of oral health and perceived oral health

**Figure 1:**
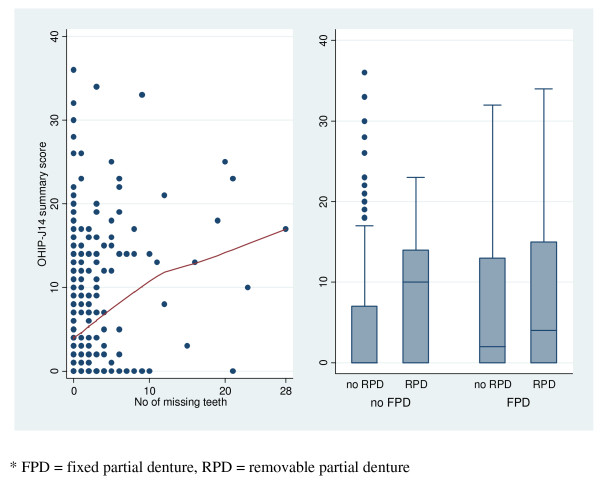
Scatter plot of OHIP-J14 scores in subjects with different number of missing teeth (including locally weighted scatter plot smoothing, lowess, that fits a flexible curve to the data) and a boxplot of OHIP-J14 scores in subjects with different denture status

The magnitude of the correlations between physical oral health characteristics and perceived oral health as measured by OHRQoL was trivial or small. The relationship between number of missing teeth and OHIP-J14 scores was nearly linear fitting a flexible curve to the data (locally weighted scatter plot smoothing, lowess) in Figure 1. Describing the linear relationship with the Pearson correlation coefficient showed a small but statistically significant correlation of 0.22 (P < 0.001). Even mean OHIP-J14 scores differed by denture status subgroups (p < 0.001, t-test, Figure 1), the point-biserial correlation coefficients between denture status (yes/no) and OHIP-J14 scores was small with 0.17 (P < 0.001).

When investigated in the levels of the two sociodemographic variables (Table 5), all subgroup correlations for number of missing teeth and OHIP-J14 were r ≤ 0.30 and all subgroup correlations for denture status and OHIP-J14 were r ≤ 0.41 - a magnitude of the correlations that would be considered "medium" according to Cohen [19].

**Table 5:** Pearson correlation coefficient between OHIP-J14 summary score and number of missing teeth and point-biserial correlation coefficient between OHIP-J14 summary score and denture status (with RPD/without RPD)

*Characteristic*		*Pearson correlation coefficient*	*P value*	*Point-biserial correlation coefficient*	*P value*
age groups					
15-24 yrs	161	0.06	0.49	0.05	0.55
25-34 yrs	285	0.05	0.35	0.10	0.10
35-44 yrs	258	0.25	0.01	0.09	0.16
45-59 yrs	207	0.25	0.01	0.15	0.03
					
Males	851	0.22	0.01	0.17	0.00
Females	60	0.27	0.04	0.24	0.06

RPD = Removable Partial Denture

## Discussion

We found good oral health, both physical, i.e., when assessed by a dentist, and perceived by the individual, in the Japan self-defense forces (JSDF). When searching the literature to compare our results, we found only a limited number of oral health reports in military populations [1,17,18]. We did not find a study assessing OHRQoL in military populations even though this concept has been increasingly recognized as an important component of health.

Our results suggest that the magnitude of correlations between physical characteristics of oral health and perceived oral health is small in this military population. However, because both "dimensions" of oral health affect the military personnel's readiness, assessment of physical and perceived health is necessary and we recommend including a measure of perceived oral health when military personnel oral health is examined. The only limited information available is on the missing tooth number of the military populations in other countries. They reported that the average number of missing teeth for the Croatian army[1] was 2.3 for 650 recruits and 5.1 for 262 professionals (all male, averaged age = 32.7 years, age range 18 to 54 years) and that for Danish military [18] was 0.02 to 0.5 (all male, average age = 25.2, age range 19 - 49 years). Our results are lower than the Croatian results and higher than the Danish results. However, the Danish subjects were younger and we found an age influence on the missing number of teeth which is also supported from studies done in civilian populations.

When compared with non-military populations, it was reported that 76.7% of the population did not have any missing tooth in New Zealand [20]. Some studies reported lower numbers such as 62.3% in India [21] and 53.5% in Israel [17]. Although direct comparison with our study findings is not possible due to the difference in age and gender distribution and tooth counting system, these data provide a general framework of how prevalent tooth loss is in the general population. Japanese population-based studies reported that 49% of subjects had intact dental arch with no missing teeth and the average number of missing teeth was 1.3 (age range 15 - 59 years) [6,22], which is higher than the result of this study.

Regarding denture status, the other key characteristic of physical oral health that we investigated, the Danish Military study (n = 223, all men, average age = 25.2 years, age range 19 - 49 years) reported no subject used removable dentures, which is lower than the current study result (4%). This might be due to the difference in the age of the studied populations. The denture status investigations in population based samples in Germany [12], Finland[23] and Malaysia[13] reported that 19% used removable partial dentures and 5% used complete dentures in Germany (average age = 43.3 years), that 18% used removable partial dentures and 12% used complete dentures in Finland (age > 30 years), and that 16.7% used removable dentures in Malaysia (age data not available). Again, a direct comparison is difficult to make because of methodological study differences. However, in absolute terms, the 4% figure of denture wearers in the JSDF is low. In the Japanese general population, the prevalence of removable denture users in the same age group as our study population is 7.0% [6] or 9.4% [22]. These numbers are substantially higher than the result of the current study.

As mentioned above, there is no report on OHRQoL in the other military populations in the literature. When compared with studies on non-military based populations, the frequencies of the impact experienced by our subjects were in general lower than previously reported. For example, the percentage of positive responses to each item ranged from 4.5 to 10.8% in a Finnish study [23] (age range 30 - 64 years), which is higher than our study results. Average OHIP14 summary scores of population-based studies in New Zealand (age 32 years old, male 51.1%) were 8.0 units [20], 5.1 - 7.7 in Sweden (age range 20 - 60 years, male 50%) [24], 7.1-7.4 in Australia (age <69 years, male 41.4%) [25], 4.7 to 5.7 in United Kingdom (age <69 years, male 45.7%) [25], and 2.4 to 4.5 in Finland (age range 30 - 64 years, male 44.3%) [23], and 11.0 in Malaysia (gender and age data not available) [13]. Once again, although direct comparison is difficult due to the age and gender differences, the OHIP14 summary score in JSDF (4.6 +/- 6.7) is low in absolute terms compared with other populations. This suggests that JSDF personnel perceive their oral health as only minimally impaired.

The significant association between missing tooth number and OHIP scores was in agreement with previous studies [2,20,25,26], which suggest a patient with more missing teeth is likely to suffer from more OHRQoL impairments. However, and also in agreement with previous studies, the correlation between the key characteristics of physical oral health and how subjects perceive their oral health is not substantial. The prevalence and severity of oral impacts also increased by usage of removable dentures, which is associated with a significant elevation of the OHIP score, as previously reported [12,23,27]. It should be noted that the number of missing teeth, which itself has a significant effect on OHRQoL, is larger in those who use removable dentures. Therefore, the presence of removable partial dentures does not necessarily cause impaired OHRQoL. It is just an indicator of impaired OHRQoL. In fact, removable dentures may improve perceived oral health in subjects with missing teeth because of its effect on oral functions such as chewing, speaking, appearance and psychosocial well-being - our study because of its cross-sectional design cannot evaluate the directionality of the denture status-OHRQoL relationship. Tooth loss' impact on OHRQoL can be compensated best with fixed partial dentures or implant dentures. When the number of teeth drops below a certain level and the tooth loss cannot be treated by fixed partial dentures, very likely the removable dentures, even if done to the highest standard in the profession and even if the dentures' quality impact on the OHRQoL[10] is maximized, cannot completely recover lost OHRQoL due to tooth loss. There is a significant cut off point of OHRQoL when a patient moves from the situation where he or she has intact dentition or missing teeth are replaced by fixed partial dentures to the situation where subjects use removable dentures [27]. The clinical implication for military personnel as well as nonmilitary subjects - is that tooth loss should be prevented as much as possible but when it happens, a major deterioration of oral health can be avoided when the magnitude of the tooth loss can still be compensated with fixed prosthodontics and extensive tooth loss, and the use of removable partial denture can be avoided.

## Conclusion

The number of missing teeth and denture status was associated with perceived oral health in the Japanese self-defense forces. Compared to Japanese civilian populations, personnel of the JSDF demonstrated good oral health.

## List of abbreviations

(OHRQoL): oral health related quality of life; (FPD): fixed partial denture; (RPD): removable partial denture; (OHIP): oral health impact profile; (OHIP-J): OHIP Japanese version; (JSDF): Japan self-defense forces; (CI): confidence interval; (SD): standard deviation

## Competing interests

The authors declare that they have no competing interests.

## Authors' contributions

YK, MTJ, KB, HT and CF conceptualized the rationale and designed the study. YK and CF contributed to the collection of data. MTJ contributed to statistical analysis and interpretation of the data. KB and YS conducted the literature review. All authors read and approved this study.

## Pre-publication history

The pre-publication history for this paper can be accessed here:

http://www.biomedcentral.com/1472-6831/11/14/prepub
